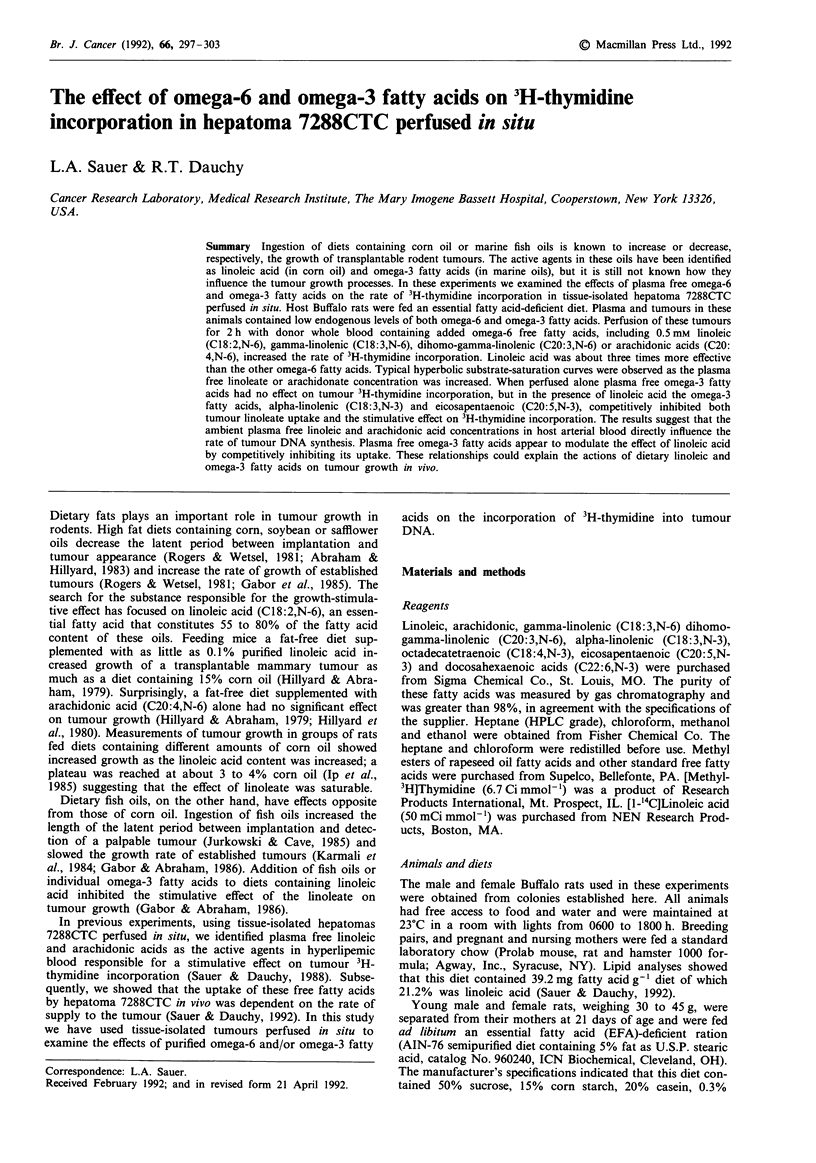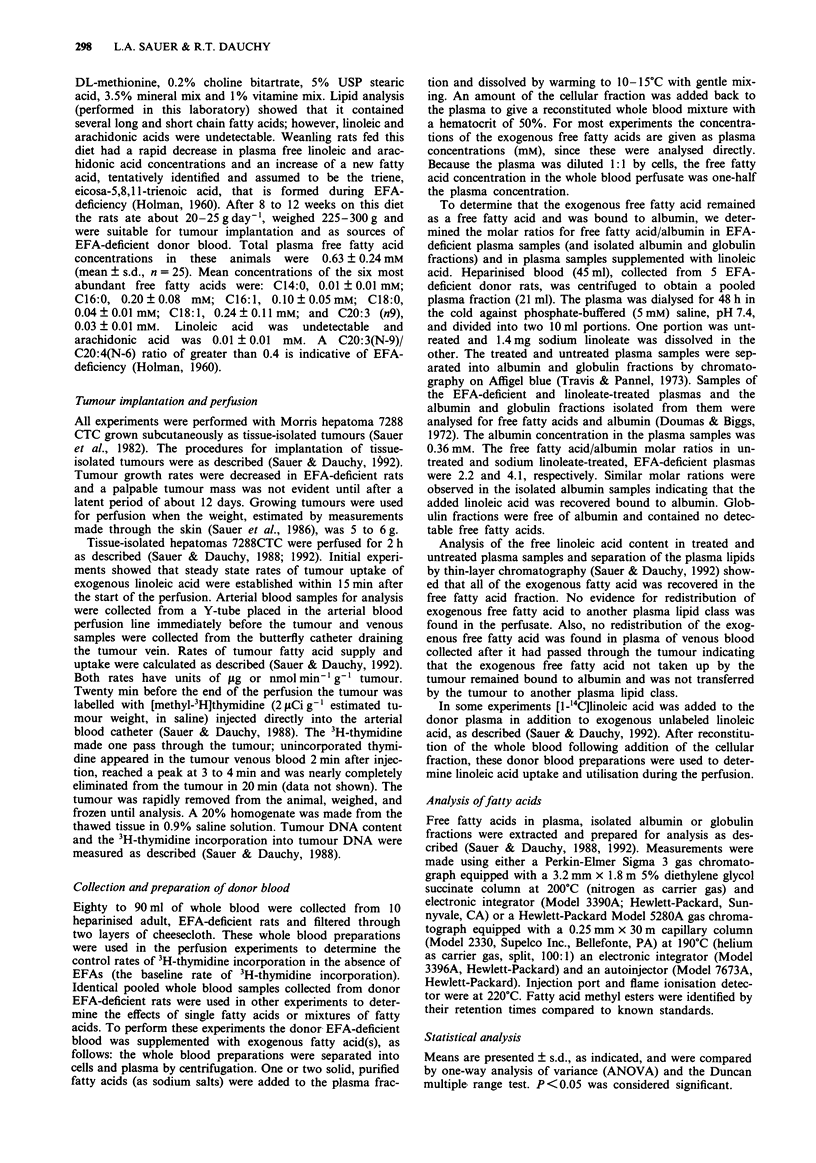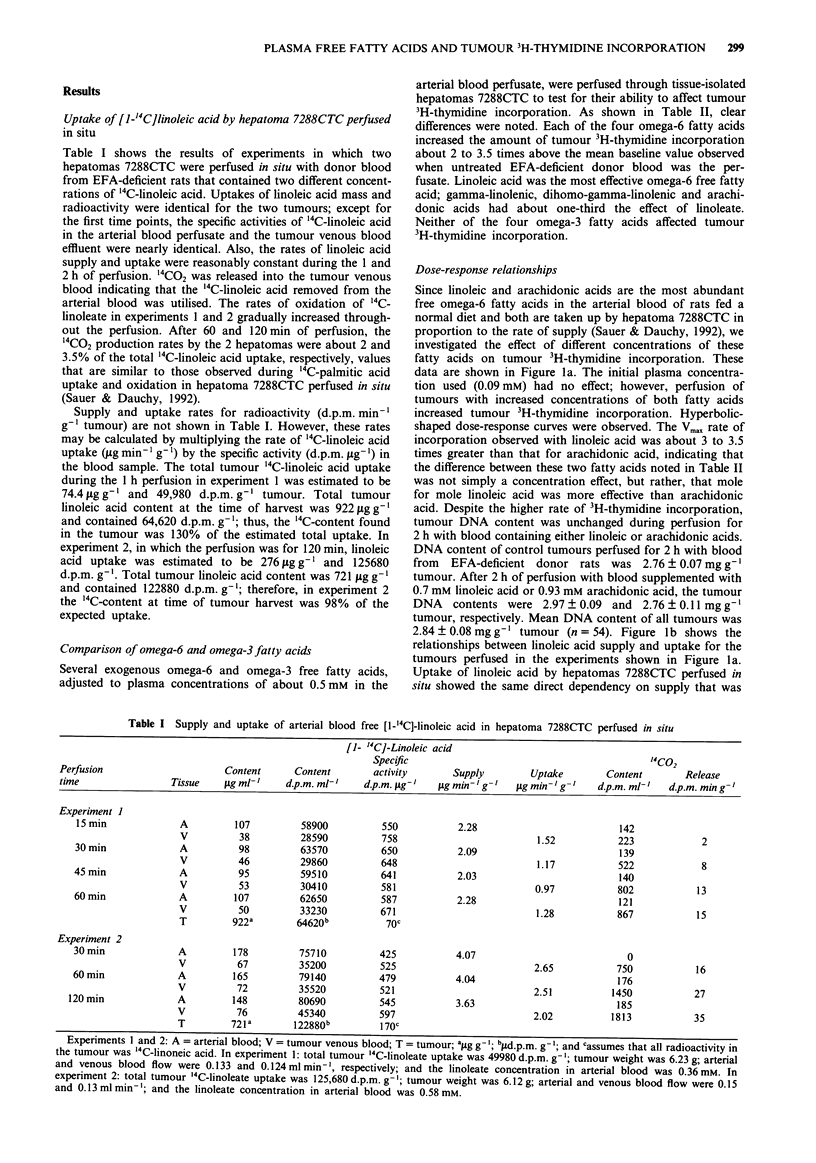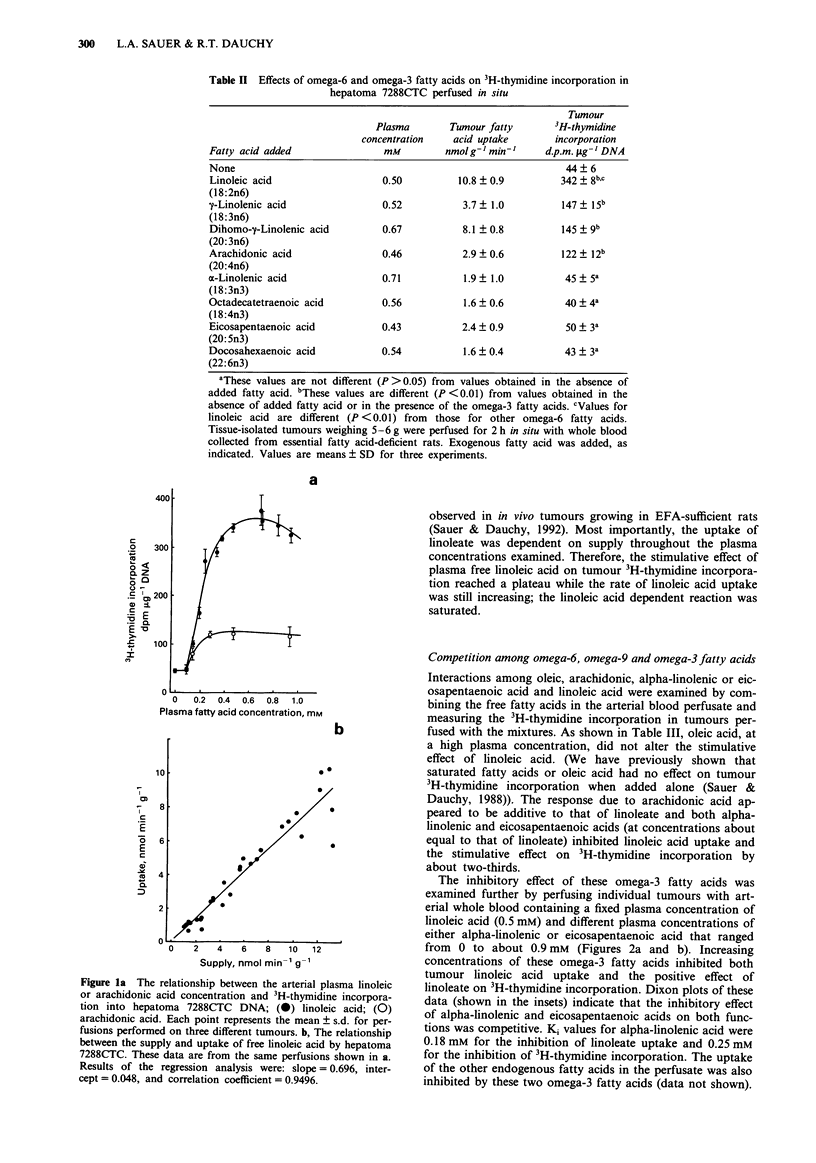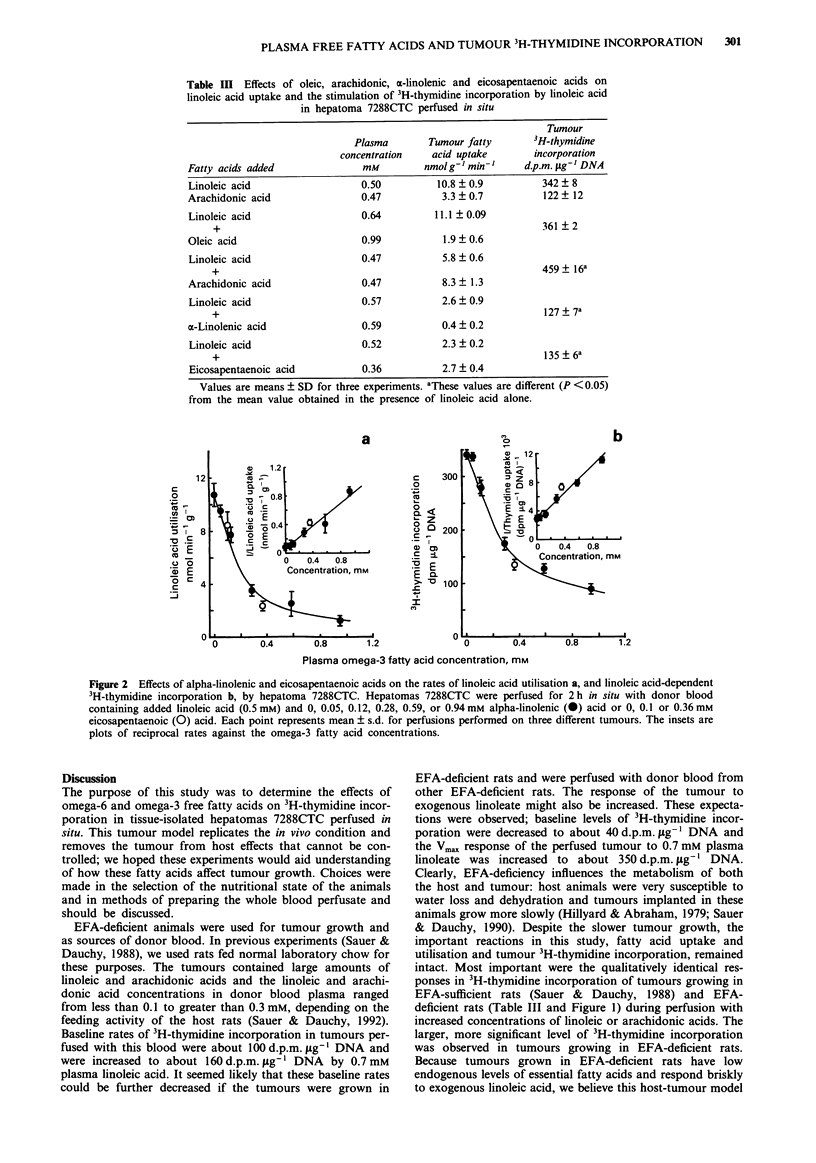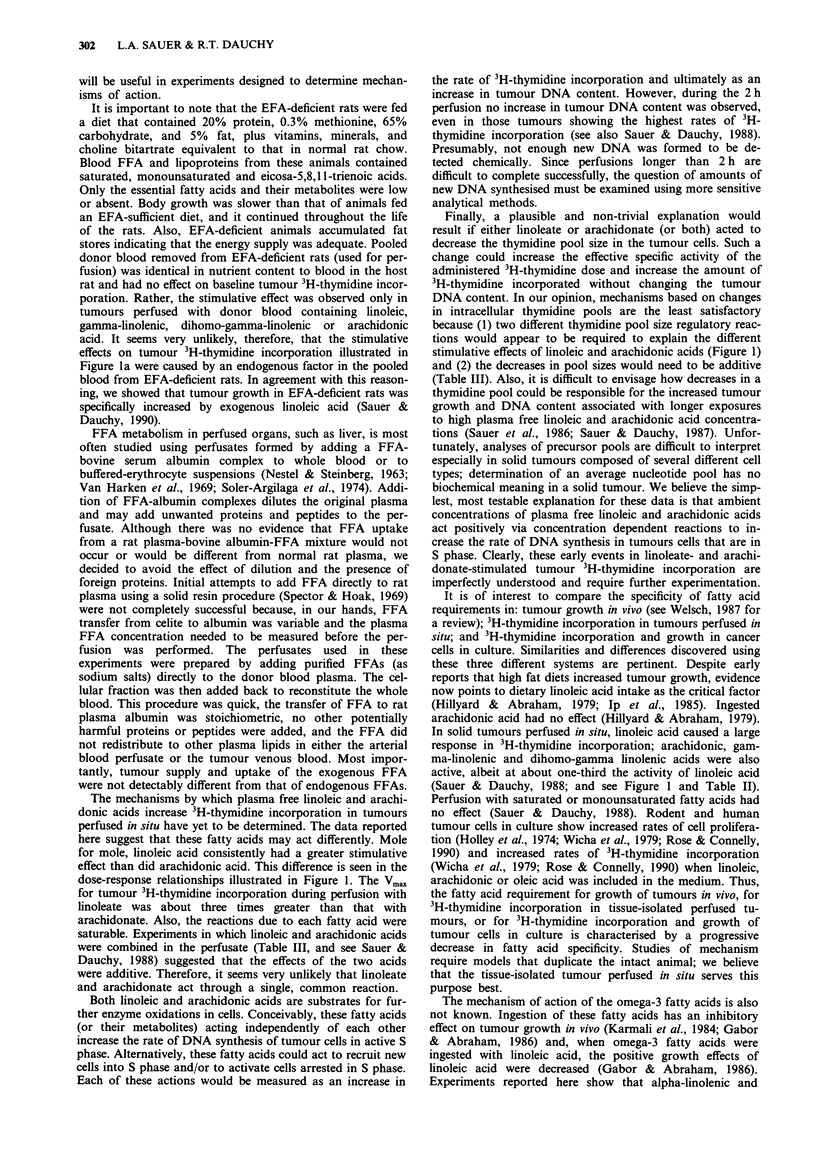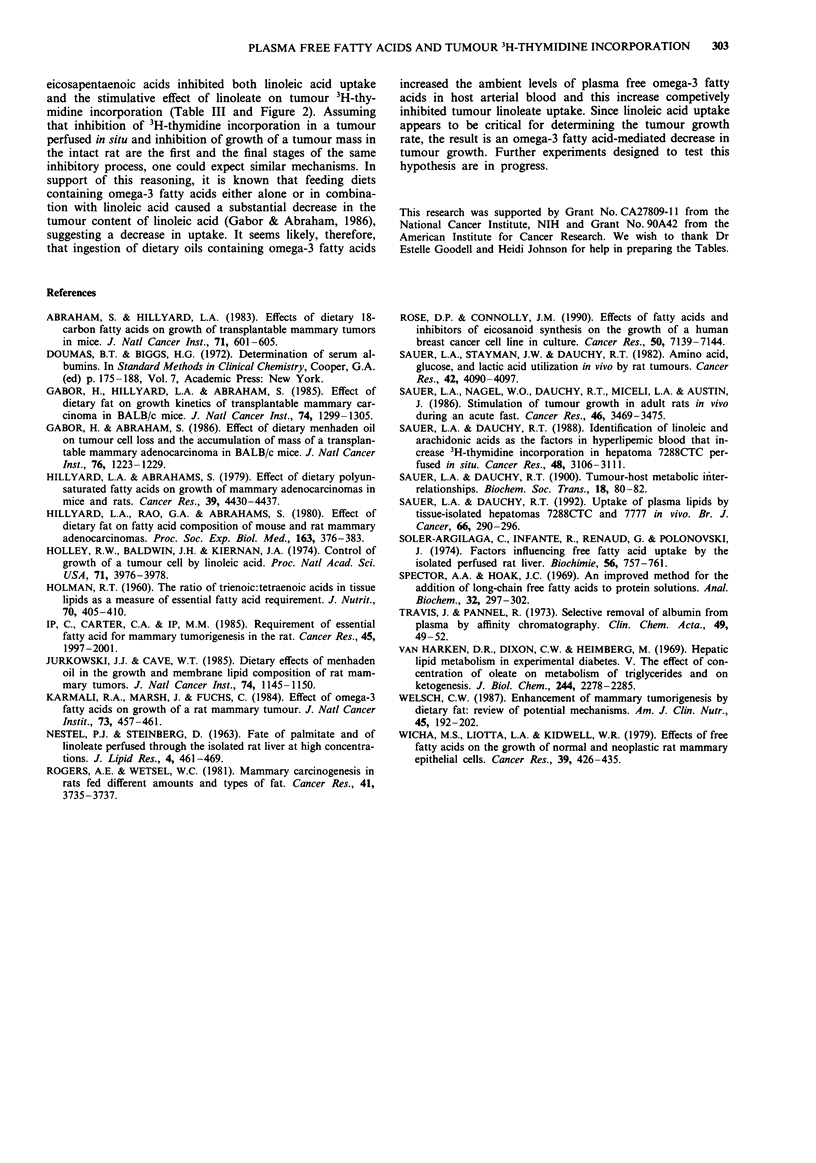# The effect of omega-6 and omega-3 fatty acids on 3H-thymidine incorporation in hepatoma 7288CTC perfused in situ.

**DOI:** 10.1038/bjc.1992.260

**Published:** 1992-08

**Authors:** L. A. Sauer, R. T. Dauchy

**Affiliations:** Cancer Research Laboratory, Mary Imogene Bassett Hospital, Cooperstown, New York 13326.

## Abstract

Ingestion of diets containing corn oil or marine fish oils is known to increase or decrease, respectively, the growth of transplantable rodent tumours. The active agents in these oils have been identified as linoleic acid (in corn oil) and omega-3 fatty acids (in marine oils), but it is still not known how they influence the tumour growth processes. In these experiments we examined the effects of plasma free omega-6 and omega-3 fatty acids on the rate of 3H-thymidine incorporation in tissue-isolated hepatoma 7288CTC perfused in situ. Host Buffalo rats were fed an essential fatty acid-deficient diet. Plasma and tumours in these animals contained low endogenous levels of both omega-6 and omega-3 fatty acids. Perfusion of these tumours for 2 h with donor whole blood containing added omega-6 free fatty acids, including 0.5 mM linoleic (C18:2,N-6), gamma-linolenic (C18:3,N-6), dihomo-gamma-linolenic (C20:3,N-6) or arachidonic acids (C20:4,N-6), increased the rate of 3H-thymidine incorporation. Linoleic acid was about three times more effective than the other omega-6 fatty acids. Typical hyperbolic substrate-saturation curves were observed as the plasma free linoleate or arachidonate concentration was increased. When perfused alone plasma free omega-3 fatty acids had no effect on tumour 3H-thymidine incorporation, but in the presence of linoleic acid the omega-3 fatty acids, alpha-linolenic (C18:3,N-3) and eicosapentaenoic (C20:5,N-3), competitively inhibited both tumour linoleate uptake and the stimulative effect on 3H-thymidine incorporation. The results suggest that the ambient plasma free linoleic and arachidonic acid concentrations in host arterial blood directly influence the rate of tumour DNA synthesis. Plasma free omega-3 fatty acids appear to modulate the effect of linoleic acid by competitively inhibiting its uptake. These relationships could explain the actions of dietary linoleic and omega-3 fatty acids on tumour growth in vivo.


Br. J. Cancer (1992), 66, 297–303 © Macmillan Press Ltd., 1992

### The effect of omega-6 and omega-3 fatty acids on $^3$H-thymidine incorporation in hepatoma 7288CTC perfused *in situ*

L.A. Sauer & R.T. Dauchy

*Cancer Research Laboratory, Medical Research Institute, The Mary Imogene Bassett Hospital, Cooperstown, New York 13326, USA.*

**Summary** Ingestion of diets containing corn oil or marine fish oils is known to increase or decrease, respectively, the growth of transplantable rodent tumours. The active agents in these oils have been identified as linoleic acid (in corn oil) and omega-3 fatty acids (in marine oils), but it is still not known how they influence the tumour growth processes. In these experiments we examined the effects of plasma free omega-6 and omega-3 fatty acids on the rate of $^3$H-thymidine incorporation in tissue-isolated hepatoma 7288CTC perfused *in situ*. Host Buffalo rats were fed an essential fatty acid-deficient diet. Plasma and tumours in these animals contained low endogenous levels of both omega-6 and omega-3 fatty acids. Perfusion of these tumours for 2 h with donor whole blood containing added omega-6 free fatty acids, including 0.5 mM linoleic (C18:2,N-6), gamma-linolenic (C18:3,N-6), dihomo-gamma-linolenic (C20:3,N-6) or arachidonic acids (C20:4,N-6), increased the rate of $^3$H-thymidine incorporation. Linoleic acid was about three times more effective than the other omega-6 fatty acids. Typical hyperbolic substrate-saturation curves were observed as the plasma free linoleate or arachidonate concentration was increased. When perfused alone plasma free omega-3 fatty acids had no effect on tumour $^3$H-thymidine incorporation, but in the presence of linoleic acid the omega-3 fatty acids, alpha-linolenic (C18:3,N-3) and eicosapentaenoic (C20:5,N-3), competitively inhibited both tumour linoleate uptake and the stimulative effect on $^3$H-thymidine incorporation. The results suggest that the ambient plasma free linoleic and arachidonic acid concentrations in host arterial blood directly influence the rate of tumour DNA synthesis. Plasma free omega-3 fatty acids appear to modulate the effect of linoleic acid by competitively inhibiting its uptake. These relationships could explain the actions of dietary linoleic and omega-3 fatty acids on tumour growth *in vivo*.

Dietary fats plays an important role in tumour growth in rodents. High fat diets containing corn, soybean or safflower oils decrease the latent period between implantation and tumour appearance (Rogers & Wetsel, 1981; Abraham & Hillyard, 1983) and increase the rate of growth of established tumours (Rogers & Wetsel, 1981; Gabor *et al.*, 1985). The search for the substance responsible for the growth-stimulative effect has focused on linoleic acid (C18:2,N-6), an essential fatty acid that constitutes 55 to 80% of the fatty acid content of these oils. Feeding mice a fat-free diet supplemented with as little as 0.1% purified linoleic acid increased growth of a transplantable mammary tumour as much as a diet containing 15% corn oil (Hillyard & Abraham, 1979). Surprisingly, a fat-free diet supplemented with arachidonic acid (C20:4,N-6) alone had no significant effect on tumour growth (Hillyard & Abraham, 1979; Hillyard *et al.*, 1980). Measurements of tumour growth in groups of rats fed diets containing different amounts of corn oil showed increased growth as the linoleic acid content was increased; a plateau was reached at about 3 to 4% corn oil (Ip *et al.*, 1985) suggesting that the effect of linoleate was saturable.

Dietary fish oils, on the other hand, have effects opposite from those of corn oil. Ingestion of fish oils increased the length of the latent period between implantation and detection of a palpable tumour (Jurkowski & Cave, 1985) and slowed the growth rate of established tumours (Karmali *et al.*, 1984; Gabor & Abraham, 1986). Addition of fish oils or individual omega-3 fatty acids to diets containing linoleic acid inhibited the stimulative effect of the linoleate on tumour growth (Gabor & Abraham, 1986).

In previous experiments, using tissue-isolated hepatomas 7288CTC perfused *in situ*, we identified plasma free linoleic and arachidonic acids as the active agents in hyperlipemic blood responsible for a stimulative effect on tumour $^3$H-thymidine incorporation (Sauer & Dauchy, 1988). Subsequently, we showed that the uptake of these free fatty acids by hepatoma 7288CTC *in vivo* was dependent on the rate of supply to the tumour (Sauer & Dauchy, 1992). In this study we have used tissue-isolated tumours perfused *in situ* to examine the effects of purified omega-6 and/or omega-3 fatty acids on the incorporation of $^3$H-thymidine into tumour DNA.

Correspondence: L.A. Sauer.
Received February 1992; and in revised form 21 April 1992.

### Materials and methods

### Reagents

Linoleic, arachidonic, gamma-linolenic (C18:3,N-6) dihomo-gamma-linolenic (C20:3,N-6), alpha-linolenic (C18:3,N-3), octadecatetraenoic (C18:4,N-3), eicosapentaenoic (C20:5,N-3) and docosahexaenoic acids (C22:6,N-3) were purchased from Sigma Chemical Co., St. Louis, MO. The purity of these fatty acids was measured by gas chromatography and was greater than 98%, in agreement with the specifications of the supplier. Heptane (HPLC grade), chloroform, methanol and ethanol were obtained from Fisher Chemical Co. The heptane and chloroform were redistilled before use. Methyl esters of rapeseed oil fatty acids and other standard free fatty acids were purchased from Supelco, Bellefonte, PA. [Methyl-$^3$H]Thymidine (6.7 Ci mmol$^{-1}$) was a product of Research Products International, Mt. Prospect, IL. [1-$^{14}$C]Linoleic acid (50 mCi mmol$^{-1}$) was purchased from NEN Research Products, Boston, MA.

### Animals and diets

The male and female Buffalo rats used in these experiments were obtained from colonies established here. All animals had free access to food and water and were maintained at 23°C in a room with lights from 0600 to 1800 h. Breeding pairs, and pregnant and nursing mothers were fed a standard laboratory chow (Prolab mouse, rat and hamster 1000 formula; Agway, Inc., Syracuse, NY). Lipid analyses showed that this diet contained 39.2 mg fatty acid g$^{-1}$ diet of which 21.2% was linoleic acid (Sauer & Dauchy, 1992).

Young male and female rats, weighing 30 to 45 g, were separated from their mothers at 21 days of age and were fed *ad libitum* an essential fatty acid (EFA)-deficient ration (AIN-76 semipurified diet containing 5% fat as U.S.P. stearic acid, catalog No. 960240, ICN Biochemical, Cleveland, OH). The manufacturer's specifications indicated that this diet contained 50% sucrose, 15% corn starch, 20% casein, 0.3%

DL-methionine, 0.2% choline bitartrate, 5% USP stearic acid, 3.5% mineral mix and 1% vitamine mix. Lipid analysis (performed in this laboratory) showed that it contained several long and short chain fatty acids; however, linoleic and arachidonic acids were undetectable. Weanling rats fed this diet had a rapid decrease in plasma free linoleic and arachidonic acid concentrations and an increase of a new fatty acid, tentatively identified and assumed to be the triene, eicosa-5,8,11-trienoic acid, that is formed during EFA-deficiency (Holman, 1960). After 8 to 12 weeks on this diet the rats ate about 20–25 g $day^{-1}$, weighed 225–300 g and were suitable for tumour implantation and as sources of EFA-deficient donor blood. Total plasma free fatty acid concentrations in these animals were $0.63 \pm 0.24$ mM (mean ± s.d., $n = 25$). Mean concentrations of the six most abundant free fatty acids were: C14:0, $0.01 \pm 0.01$ mM; C16:0, $0.20 \pm 0.08$ mM; C16:1, $0.10 \pm 0.05$ mM; C18:0, $0.04 \pm 0.01$ mM; C18:1, $0.24 \pm 0.11$ mM; and C20:3 (*n*9), $0.03 \pm 0.01$ mM. Linoleic acid was undetectable and arachidonic acid was $0.01 \pm 0.01$ mM. A C20:3(N-9)/C20:4(N-6) ratio of greater than 0.4 is indicative of EFA-deficiency (Holman, 1960).

*Tumour implantation and perfusion*

All experiments were performed with Morris hepatoma 7288 CTC grown subcutaneously as tissue-isolated tumours (Sauer *et al.*, 1982). The procedures for implantation of tissue-isolated tumours were as described (Sauer & Dauchy, 1992). Tumour growth rates were decreased in EFA-deficient rats and a palpable tumour mass was not evident until after a latent period of about 12 days. Growing tumours were used for perfusion when the weight, estimated by measurements made through the skin (Sauer *et al.*, 1986), was 5 to 6 g.

Tissue-isolated hepatomas 7288CTC were perfused for 2 h as described (Sauer & Dauchy, 1988; 1992). Initial experiments showed that steady state rates of tumour uptake of exogenous linoleic acid were established within 15 min after the start of the perfusion. Arterial blood samples for analysis were collected from a Y-tube placed in the arterial blood perfusion line immediately before the tumour and venous samples were collected from the butterfly catheter draining the tumour vein. Rates of tumour fatty acid supply and uptake were calculated as described (Sauer & Dauchy, 1992). Both rates have units of $\mu g$ or nmol $min^{-1}$ $g^{-1}$ tumour. Twenty min before the end of the perfusion the tumour was labelled with [methyl-$^{3}$H]thymidine (2 $\mu Ci$ $g^{-1}$ estimated tumour weight, in saline) injected directly into the arterial blood catheter (Sauer & Dauchy, 1988). The $^{3}$H-thymidine made one pass through the tumour; unincorporated thymidine appeared in the tumour venous blood 2 min after injection, reached a peak at 3 to 4 min and was nearly completely eliminated from the tumour in 20 min (data not shown). The tumour was rapidly removed from the animal, weighed, and frozen until analysis. A 20% homogenate was made from the thawed tissue in 0.9% saline solution. Tumour DNA content and the $^{3}$H-thymidine incorporation into tumour DNA were measured as described (Sauer & Dauchy, 1988).

*Collection and preparation of donor blood*

Eighty to 90 ml of whole blood were collected from 10 heparinised adult, EFA-deficient rats and filtered through two layers of cheesecloth. These whole blood preparations were used in the perfusion experiments to determine the control rates of $^{3}$H-thymidine incorporation in the absence of EFAs (the baseline rate of $^{3}$H-thymidine incorporation). Identical pooled whole blood samples collected from donor EFA-deficient rats were used in other experiments to determine the effects of single fatty acids or mixtures of fatty acids. To perform these experiments the donor EFA-deficient blood was supplemented with exogenous fatty acid(s), as follows: the whole blood preparations were separated into cells and plasma by centrifugation. One or two solid, purified fatty acids (as sodium salts) were added to the plasma fraction and dissolved by warming to 10–15°C with gentle mixing. An amount of the cellular fraction was added back to the plasma to give a reconstituted whole blood mixture with a hematocrit of 50%. For most experiments the concentrations of the exogenous free fatty acids are given as plasma concentrations (mM), since these were analysed directly. Because the plasma was diluted 1:1 by cells, the free fatty acid concentration in the whole blood perfusate was one-half the plasma concentration.

To determine that the exogenous free fatty acid remained as a free fatty acid and was bound to albumin, we determined the molar ratios for free fatty acid/albumin in EFA-deficient plasma samples (and isolated albumin and globulin fractions) and in plasma samples supplemented with linoleic acid. Heparinised blood (45 ml), collected from 5 EFA-deficient donor rats, was centrifuged to obtain a pooled plasma fraction (21 ml). The plasma was dialysed for 48 h in the cold against phosphate-buffered (5 mM) saline, pH 7.4, and divided into two 10 ml portions. One portion was untreated and 1.4 mg sodium linoleate was dissolved in the other. The treated and untreated plasma samples were separated into albumin and globulin fractions by chromatography on Affigel blue (Travis & Pannel, 1973). Samples of the EFA-deficient and linoleate-treated plasmas and the albumin and globulin fractions isolated from them were analysed for free fatty acids and albumin (Doumas & Biggs, 1972). The albumin concentration in the plasma samples was 0.36 mM. The free fatty acid/albumin molar ratios in untreated and sodium linoleate-treated, EFA-deficient plasmas were 2.2 and 4.1, respectively. Similar molar rations were observed in the isolated albumin samples indicating that the added linoleic acid was recovered bound to albumin. Globulin fractions were free of albumin and contained no detectable free fatty acids.

Analysis of the free linoleic acid content in treated and untreated plasma samples and separation of the plasma lipids by thin-layer chromatography (Sauer & Dauchy, 1992) showed that all of the exogenous fatty acid was recovered in the free fatty acid fraction. No evidence for redistribution of exogenous free fatty acid to another plasma lipid class was found in the perfusate. Also, no redistribution of the exogenous free fatty acid was found in plasma of venous blood collected after it had passed through the tumour indicating that the exogenous free fatty acid not taken up by the tumour remained bound to albumin and was not transferred by the tumour to another plasma lipid class.

In some experiments [1-$^{14}$C]linoleic acid was added to the donor plasma in addition to exogenous unlabeled linoleic acid, as described (Sauer & Dauchy, 1992). After reconstitution of the whole blood following addition of the cellular fraction, these donor blood preparations were used to determine linoleic acid uptake and utilisation during the perfusion.

*Analysis of fatty acids*

Free fatty acids in plasma, isolated albumin or globulin fractions were extracted and prepared for analysis as described (Sauer & Dauchy, 1988, 1992). Measurements were made using either a Perkin-Elmer Sigma 3 gas chromatograph equipped with a 3.2 mm × 1.8 m 5% diethylene glycol succinate column at 200°C (nitrogen as carrier gas) and electronic integrator (Model 3390A; Hewlett-Packard, Sunnyvale, CA) or a Hewlett-Packard Model 5280A gas chromatograph equipped with a 0.25 mm × 30 m capillary column (Model 2330, Supelco Inc., Bellefonte, PA) at 190°C (helium as carrier gas, split, 100:1) an electronic integrator (Model 3396A, Hewlett-Packard) and an autoinjector (Model 7673A, Hewlett-Packard). Injection port and flame ionisation detector were at 220°C. Fatty acid methyl esters were identified by their retention times compared to known standards.

*Statistical analysis*

Means are presented ± s.d., as indicated, and were compared by one-way analysis of variance (ANOVA) and the Duncan multiple range test. $P < 0.05$ was considered significant.

### Results

*Uptake of [1-$^{14}$C]linoleic acid by hepatoma 7288CTC perfused* in situ

Table I shows the results of experiments in which two hepatomas 7288CTC were perfused *in situ* with donor blood from EFA-deficient rats that contained two different concentrations of $^{14}$C-linoleic acid. Uptakes of linoleic acid mass and radioactivity were identical for the two tumours; except for the first time points, the specific activities of $^{14}$C-linoleic acid in the arterial blood perfusate and the tumour venous blood effluent were nearly identical. Also, the rates of linoleic acid supply and uptake were reasonably constant during the 1 and 2 h of perfusion. $^{14}CO_2$ was released into the tumour venous blood indicating that the $^{14}$C-linoleic acid removed from the arterial blood was utilised. The rates of oxidation of $^{14}$C-linoleate in experiments 1 and 2 gradually increased throughout the perfusion. After 60 and 120 min of perfusion, the $^{14}CO_2$ production rates by the 2 hepatomas were about 2 and 3.5% of the total $^{14}$C-linoleic acid uptake, respectively, values that are similar to those observed during $^{14}$C-palmitic acid uptake and oxidation in hepatoma 7288CTC perfused *in situ* (Sauer & Dauchy, 1992).

Supply and uptake rates for radioactivity (d.p.m. min$^{-1}$ g$^{-1}$ tumour) are not shown in Table I. However, these rates may be calculated by multiplying the rate of $^{14}$C-linoleic acid uptake ($\mu$g min$^{-1}$ g$^{-1}$) by the specific activity (d.p.m. $\mu$g$^{-1}$) in the blood sample. The total tumour $^{14}$C-linoleic acid uptake during the 1 h perfusion in experiment 1 was estimated to be 74.4 $\mu$g g$^{-1}$ and 49,980 d.p.m. g$^{-1}$ tumour. Total tumour linoleic acid content at the time of harvest was 922 $\mu$g g$^{-1}$ and contained 64,620 d.p.m. g$^{-1}$; thus, the $^{14}$C-content found in the tumour was 130% of the estimated total uptake. In experiment 2, in which the perfusion was for 120 min, linoleic acid uptake was estimated to be 276 $\mu$g g$^{-1}$ and 125680 d.p.m. g$^{-1}$. Total tumour linoleic acid content was 721 $\mu$g g$^{-1}$ and contained 122880 d.p.m. g$^{-1}$; therefore, in experiment 2 the $^{14}$C-content at time of tumour harvest was 98% of the expected uptake.

*Comparison of omega-6 and omega-3 fatty acids*

Several exogenous omega-6 and omega-3 free fatty acids, adjusted to plasma concentrations of about 0.5 mM in the arterial blood perfusate, were perfused through tissue-isolated hepatomas 7288CTC to test for their ability to affect tumour $^3$H-thymidine incorporation. As shown in Table II, clear differences were noted. Each of the four omega-6 fatty acids increased the amount of tumour $^3$H-thymidine incorporation about 2 to 3.5 times above the mean baseline value observed when untreated EFA-deficient donor blood was the perfusate. Linoleic acid was the most effective omega-6 free fatty acid; gamma-linolenic, dihomo-gamma-linolenic and arachidonic acids had about one-third the effect of linoleate. Neither of the four omega-3 fatty acids affected tumour $^3$H-thymidine incorporation.

*Dose-response relationships*

Since linoleic and arachidonic acids are the most abundant free omega-6 fatty acids in the arterial blood of rats fed a normal diet and both are taken up by hepatoma 7288CTC in proportion to the rate of supply (Sauer & Dauchy, 1992), we investigated the effect of different concentrations of these fatty acids on tumour $^3$H-thymidine incorporation. These data are shown in Figure 1a. The initial plasma concentration used (0.09 mM) had no effect; however, perfusion of tumours with increased concentrations of both fatty acids increased tumour $^3$H-thymidine incorporation. Hyperbolic-shaped dose-response curves were observed. The $V_{max}$ rate of incorporation observed with linoleic acid was about 3 to 3.5 times greater than that for arachidonic acid, indicating that the difference between these two fatty acids noted in Table II was not simply a concentration effect, but rather, that mole for mole linoleic acid was more effective than arachidonic acid. Despite the higher rate of $^3$H-thymidine incorporation, tumour DNA content was unchanged during perfusion for 2 h with blood containing either linoleic or arachidonic acids. DNA content of control tumours perfused for 2 h with blood from EFA-deficient donor rats was 2.76 ± 0.07 mg g$^{-1}$ tumour. After 2 h of perfusion with blood supplemented with 0.7 mM linoleic acid or 0.93 mM arachidonic acid, the tumour DNA contents were 2.97 ± 0.09 and 2.76 ± 0.11 mg g$^{-1}$ tumour, respectively. Mean DNA content of all tumours was 2.84 ± 0.08 mg g$^{-1}$ tumour ($n = 54$). Figure 1b shows the relationships between linoleic acid supply and uptake for the tumours perfused in the experiments shown in Figure 1a. Uptake of linoleic acid by hepatomas 7288CTC perfused *in situ* showed the same direct dependency on supply that was

**Table I** Supply and uptake of arterial blood free [1-$^{14}$C]-linoleic acid in hepatoma 7288CTC perfused *in situ*

| | | [1-$^{14}$C]-Linoleic acid | | | | | $^{14}CO_2$ | |
|---|---|---|---|---|---|---|---|---|
| *Perfusion time* | *Tissue* | *Content μg ml$^{-1}$* | *Content d.p.m. ml$^{-1}$* | *Specific activity d.p.m. μg$^{-1}$* | *Supply μg min$^{-1}$ g$^{-1}$* | *Uptake μg min$^{-1}$ g$^{-1}$* | *Content d.p.m. ml$^{-1}$* | *Release d.p.m. min g$^{-1}$* |
| *Experiment 1* | | | | | | | | |
| 15 min | A | 107 | 58900 | 550 | 2.28 | | 142 | |
| | V | 38 | 28590 | 758 | | 1.52 | 223 | 2 |
| 30 min | A | 98 | 63570 | 650 | 2.09 | | 139 | |
| | V | 46 | 29860 | 648 | | 1.17 | 522 | 8 |
| 45 min | A | 95 | 59510 | 641 | 2.03 | | 140 | |
| | V | 53 | 30410 | 581 | | 0.97 | 802 | 13 |
| 60 min | A | 107 | 62650 | 587 | 2.28 | | 121 | |
| | V | 50 | 33230 | 671 | | 1.28 | 867 | 15 |
| | T | 922[a] | 64620[b] | 70[c] | | | | |
| *Experiment 2* | | | | | | | | |
| 30 min | A | 178 | 75710 | 425 | 4.07 | | 0 | |
| | V | 67 | 35200 | 525 | | 2.65 | 750 | 16 |
| 60 min | A | 165 | 79140 | 479 | 4.04 | | 176 | |
| | V | 72 | 35520 | 521 | | 2.51 | 1450 | 27 |
| 120 min | A | 148 | 80690 | 545 | 3.63 | | 185 | |
| | V | 76 | 45340 | 597 | | 2.02 | 1813 | 35 |
| | T | 721[a] | 122880[b] | 170[c] | | | | |

Experiments 1 and 2: A = arterial blood; V = tumour venous blood; T = tumour; [a]μg g$^{-1}$; [b]μd.p.m. g$^{-1}$; and [c]assumes that all radioactivity in the tumour was $^{14}$C-linoneic acid. In experiment 1: total tumour $^{14}$C-linoleate uptake was 49980 d.p.m. g$^{-1}$; tumour weight was 6.23 g; arterial and venous blood flow were 0.133 and 0.124 ml min$^{-1}$, respectively; and the linoleate concentration in arterial blood was 0.36 mM. In experiment 2: total tumour $^{14}$C-linoleate uptake was 125,680 d.p.m. g$^{-1}$; tumour weight was 6.12 g; arterial and venous blood flow were 0.15 and 0.13 ml min$^{-1}$; and the linoleate concentration in arterial blood was 0.58 mM.

**Table II** Effects of omega-6 and omega-3 fatty acids on $^3$H-thymidine incorporation in hepatoma 7288CTC perfused *in situ*

| *Fatty acid added* | *Plasma concentration* mM | *Tumour fatty acid uptake* nmol $g^{-1}$ $min^{-1}$ | *Tumour $^3$H-thymidine incorporation* d.p.m. $\mu g^{-1}$ DNA |
|---|---|---|---|
| None | | | 44 ± 6 |
| Linoleic acid (18:2n6) | 0.50 | 10.8 ± 0.9 | 342 ± 8[b,c] |
| γ-Linolenic acid (18:3n6) | 0.52 | 3.7 ± 1.0 | 147 ± 15[b] |
| Dihomo-γ-Linolenic acid (20:3n6) | 0.67 | 8.1 ± 0.8 | 145 ± 9[b] |
| Arachidonic acid (20:4n6) | 0.46 | 2.9 ± 0.6 | 122 ± 12[b] |
| α-Linolenic acid (18:3n3) | 0.71 | 1.9 ± 1.0 | 45 ± 5[a] |
| Octadecatetraenoic acid (18:4n3) | 0.56 | 1.6 ± 0.6 | 40 ± 4[a] |
| Eicosapentaenoic acid (20:5n3) | 0.43 | 2.4 ± 0.9 | 50 ± 3[a] |
| Docosahexaenoic acid (22:6n3) | 0.54 | 1.6 ± 0.4 | 43 ± 3[a] |

[a]These values are not different ($P > 0.05$) from values obtained in the absence of added fatty acid. [b]These values are different ($P < 0.01$) from values obtained in the absence of added fatty acid or in the presence of the omega-3 fatty acids. [c]Values for linoleic acid are different ($P < 0.01$) from those for other omega-6 fatty acids. Tissue-isolated tumours weighing 5–6 g were perfused for 2 h *in situ* with whole blood collected from essential fatty acid-deficient rats. Exogenous fatty acid was added, as indicated. Values are means ± SD for three experiments.

**Figure 1a** The relationship between the arterial plasma linoleic or arachidonic acid concentration and $^3$H-thymidine incorporation into hepatoma 7288CTC DNA; (●) linoleic acid; (○) arachidonic acid. Each point represents the mean ± s.d. for perfusions performed on three different tumours. **b**, The relationship between the supply and uptake of free linoleic acid by hepatoma 7288CTC. These data are from the same perfusions shown in **a**. Results of the regression analysis were: slope = 0.696, intercept = 0.048, and correlation coefficient = 0.9496.

observed in *in vivo* tumours growing in EFA-sufficient rats (Sauer & Dauchy, 1992). Most importantly, the uptake of linoleate was dependent on supply throughout the plasma concentrations examined. Therefore, the stimulative effect of plasma free linoleic acid on tumour $^3$H-thymidine incorporation reached a plateau while the rate of linoleic acid uptake was still increasing; the linoleic acid dependent reaction was saturated.

*Competition among omega-6, omega-9 and omega-3 fatty acids*

Interactions among oleic, arachidonic, alpha-linolenic or eicosapentaenoic acid and linoleic acid were examined by combining the free fatty acids in the arterial blood perfusate and measuring the $^3$H-thymidine incorporation in tumours perfused with the mixtures. As shown in Table III, oleic acid, at a high plasma concentration, did not alter the stimulative effect of linoleic acid. (We have previously shown that saturated fatty acids or oleic acid had no effect on tumour $^3$H-thymidine incorporation when added alone (Sauer & Dauchy, 1988)). The response due to arachidonic acid appeared to be additive to that of linoleate and both alpha-linolenic and eicosapentaenoic acids (at concentrations about equal to that of linoleate) inhibited linoleic acid uptake and the stimulative effect on $^3$H-thymidine incorporation by about two-thirds.

The inhibitory effect of these omega-3 fatty acids was examined further by perfusing individual tumours with arterial whole blood containing a fixed plasma concentration of linoleic acid (0.5 mM) and different plasma concentrations of either alpha-linolenic or eicosapentaenoic acid that ranged from 0 to about 0.9 mM (Figures 2a and b). Increasing concentrations of these omega-3 fatty acids inhibited both tumour linoleic acid uptake and the positive effect of linoleate on $^3$H-thymidine incorporation. Dixon plots of these data (shown in the insets) indicate that the inhibitory effect of alpha-linolenic and eicosapentaenoic acids on both functions was competitive. $K_i$ values for alpha-linolenic acid were 0.18 mM for the inhibition of linoleate uptake and 0.25 mM for the inhibition of $^3$H-thymidine incorporation. The uptake of the other endogenous fatty acids in the perfusate was also inhibited by these two omega-3 fatty acids (data not shown).

**Table III** Effects of oleic, arachidonic, α-linolenic and eicosapentaenoic acids on linoleic acid uptake and the stimulation of $^3$H-thymidine incorporation by linoleic acid in hepatoma 7288CTC perfused *in situ*

| *Fatty acids added* | *Plasma concentration* mM | *Tumour fatty acid uptake* nmol $g^{-1}$ $min^{-1}$ | *Tumour* $^3$*H-thymidine incorporation* d.p.m. $\mu g^{-1}$ DNA |
|---|---|---|---|
| Linoleic acid | 0.50 | 10.8 ± 0.9 | 342 ± 8 |
| Arachidonic acid | 0.47 | 3.3 ± 0.7 | 122 ± 12 |
| Linoleic acid | 0.64 | 11.1 ± 0.09 | |
| + | | | 361 ± 2 |
| Oleic acid | 0.99 | 1.9 ± 0.6 | |
| Linoleic acid | 0.47 | 5.8 ± 0.6 | |
| + | | | 459 ± 16[a] |
| Arachidonic acid | 0.47 | 8.3 ± 1.3 | |
| Linoleic acid | 0.57 | 2.6 ± 0.9 | |
| + | | | 127 ± 7[a] |
| α-Linolenic acid | 0.59 | 0.4 ± 0.2 | |
| Linoleic acid | 0.52 | 2.3 ± 0.2 | |
| + | | | 135 ± 6[a] |
| Eicosapentaenoic acid | 0.36 | 2.7 ± 0.4 | |

Values are means ± SD for three experiments. [a]These values are different ($P < 0.05$) from the mean value obtained in the presence of linoleic acid alone.

**Figure 2** Effects of alpha-linolenic and eicosapentaenoic acids on the rates of linoleic acid utilisation **a**, and linoleic acid-dependent $^3$H-thymidine incorporation **b**, by hepatoma 7288CTC. Hepatomas 7288CTC were perfused for 2 h *in situ* with donor blood containing added linoleic acid (0.5 mM) and 0, 0.05, 0.12, 0.28, 0.59, or 0.94 mM alpha-linolenic (●) acid or 0, 0.1 or 0.36 mM eicosapentaenoic (○) acid. Each point represents mean ± s.d. for perfusions performed on three different tumours. The insets are plots of reciprocal rates against the omega-3 fatty acid concentrations.

### Discussion

The purpose of this study was to determine the effects of omega-6 and omega-3 free fatty acids on $^3$H-thymidine incorporation in tissue-isolated hepatomas 7288CTC perfused *in situ*. This tumour model replicates the *in vivo* condition and removes the tumour from host effects that cannot be controlled; we hoped these experiments would aid understanding of how these fatty acids affect tumour growth. Choices were made in the selection of the nutritional state of the animals and in methods of preparing the whole blood perfusate and should be discussed.

EFA-deficient animals were used for tumour growth and as sources of donor blood. In previous experiments (Sauer & Dauchy, 1988), we used rats fed normal laboratory chow for these purposes. The tumours contained large amounts of linoleic and arachidonic acids and the linoleic and arachidonic acid concentrations in donor blood plasma ranged from less than 0.1 to greater than 0.3 mM, depending on the feeding activity of the host rats (Sauer & Dauchy, 1992). Baseline rates of $^3$H-thymidine incorporation in tumours perfused with this blood were about 100 d.p.m. $\mu g^{-1}$ DNA and were increased to about 160 d.p.m. $\mu g^{-1}$ DNA by 0.7 mM plasma linoleic acid. It seemed likely that these baseline rates could be further decreased if the tumours were grown in EFA-deficient rats and were perfused with donor blood from other EFA-deficient rats. The response of the tumour to exogenous linoleate might also be increased. These expectations were observed; baseline levels of $^3$H-thymidine incorporation were decreased to about 40 d.p.m. $\mu g^{-1}$ DNA and the $V_{max}$ response of the perfused tumour to 0.7 mM plasma linoleate was increased to about 350 d.p.m. $\mu g^{-1}$ DNA. Clearly, EFA-deficiency influences the metabolism of both the host and tumour: host animals were very susceptible to water loss and dehydration and tumours implanted in these animals grow more slowly (Hillyard & Abraham, 1979; Sauer & Dauchy, 1990). Despite the slower tumour growth, the important reactions in this study, fatty acid uptake and utilisation and tumour $^3$H-thymidine incorporation, remained intact. Most important were the qualitatively identical responses in $^3$H-thymidine incorporation of tumours growing in EFA-sufficient rats (Sauer & Dauchy, 1988) and EFA-deficient rats (Table III and Figure 1) during perfusion with increased concentrations of linoleic or arachidonic acids. The larger, more significant level of $^3$H-thymidine incorporation was observed in tumours growing in EFA-deficient rats. Because tumours grown in EFA-deficient rats have low endogenous levels of essential fatty acids and respond briskly to exogenous linoleic acid, we believe this host-tumour model will be useful in experiments designed to determine mechanisms of action.

It is important to note that the EFA-deficient rats were fed a diet that contained 20% protein, 0.3% methionine, 65% carbohydrate, and 5% fat, plus vitamins, minerals, and choline bitartrate equivalent to that in normal rat chow. Blood FFA and lipoproteins from these animals contained saturated, monounsaturated and eicosa-5,8,11-trienoic acids. Only the essential fatty acids and their metabolites were low or absent. Body growth was slower than that of animals fed an EFA-sufficient diet, and it continued throughout the life of the rats. Also, EFA-deficient animals accumulated fat stores indicating that the energy supply was adequate. Pooled donor blood removed from EFA-deficient rats (used for perfusion) was identical in nutrient content to blood in the host rat and had no effect on baseline tumour $^3$H-thymidine incorporation. Rather, the stimulative effect was observed only in tumours perfused with donor blood containing linoleic, gamma-linolenic, dihomo-gamma-linolenic or arachidonic acid. It seems very unlikely, therefore, that the stimulative effects on tumour $^3$H-thymidine incorporation illustrated in Figure 1a were caused by an endogenous factor in the pooled blood from EFA-deficient rats. In agreement with this reasoning, we showed that tumour growth in EFA-deficient rats was specifically increased by exogenous linoleic acid (Sauer & Dauchy, 1990).

FFA metabolism in perfused organs, such as liver, is most often studied using perfusates formed by adding a FFA-bovine serum albumin complex to whole blood or to buffered-erythrocyte suspensions (Nestel & Steinberg, 1963; Van Harken *et al.*, 1969; Soler-Argilaga *et al.*, 1974). Addition of FFA-albumin complexes dilutes the original plasma and may add unwanted proteins and peptides to the perfusate. Although there was no evidence that FFA uptake from a rat plasma-bovine albumin-FFA mixture would not occur or would be different from normal rat plasma, we decided to avoid the effect of dilution and the presence of foreign proteins. Initial attempts to add FFA directly to rat plasma using a solid resin procedure (Spector & Hoak, 1969) were not completely successful because, in our hands, FFA transfer from celite to albumin was variable and the plasma FFA concentration needed to be measured before the perfusion was performed. The perfusates used in these experiments were prepared by adding purified FFAs (as sodium salts) directly to the donor blood plasma. The cellular fraction was then added back to reconstitute the whole blood. This procedure was quick, the transfer of FFA to rat plasma albumin was stoichiometric, no other potentially harmful proteins or peptides were added, and the FFA did not redistribute to other plasma lipids in either the arterial blood perfusate or the tumour venous blood. Most importantly, tumour supply and uptake of the exogenous FFA were not detectably different from that of endogenous FFAs.

The mechanisms by which plasma free linoleic and arachidonic acids increase $^3$H-thymidine incorporation in tumours perfused *in situ* have yet to be determined. The data reported here suggest that these fatty acids may act differently. Mole for mole, linoleic acid consistently had a greater stimulative effect than did arachidonic acid. This difference is seen in the dose-response relationships illustrated in Figure 1. The $V_{max}$ for tumour $^3$H-thymidine incorporation during perfusion with linoleate was about three times greater than that with arachidonate. Also, the reactions due to each fatty acid were saturable. Experiments in which linoleic and arachidonic acids were combined in the perfusate (Table III, and see Sauer & Dauchy, 1988) suggested that the effects of the two acids were additive. Therefore, it seems very unlikely that linoleate and arachidonate act through a single, common reaction.

Both linoleic and arachidonic acids are substrates for further enzyme oxidations in cells. Conceivably, these fatty acids (or their metabolites) acting independently of each other increase the rate of DNA synthesis of tumour cells in active S phase. Alternatively, these fatty acids could act to recruit new cells into S phase and/or to activate cells arrested in S phase. Each of these actions would be measured as an increase in the rate of $^3$H-thymidine incorporation and ultimately as an increase in tumour DNA content. However, during the 2 h perfusion no increase in tumour DNA content was observed, even in those tumours showing the highest rates of $^3$H-thymidine incorporation (see also Sauer & Dauchy, 1988). Presumably, not enough new DNA was formed to be detected chemically. Since perfusions longer than 2 h are difficult to complete successfully, the question of amounts of new DNA synthesised must be examined using more sensitive analytical methods.

Finally, a plausible and non-trivial explanation would result if either linoleate or arachidonate (or both) acted to decrease the thymidine pool size in the tumour cells. Such a change could increase the effective specific activity of the administered $^3$H-thymidine dose and increase the amount of $^3$H-thymidine incorporated without changing the tumour DNA content. In our opinion, mechanisms based on changes in intracellular thymidine pools are the least satisfactory because (1) two different thymidine pool size regulatory reactions would appear to be required to explain the different stimulative effects of linoleic and arachidonic acids (Figure 1) and (2) the decreases in pool sizes would need to be additive (Table III). Also, it is difficult to envisage how decreases in a thymidine pool could be responsible for the increased tumour growth and DNA content associated with longer exposures to high plasma free linoleic and arachidonic acid concentrations (Sauer *et al.*, 1986; Sauer & Dauchy, 1987). Unfortunately, analyses of precursor pools are difficult to interpret especially in solid tumours composed of several different cell types; determination of an average nucleotide pool has no biochemical meaning in a solid tumour. We believe the simplest, most testable explanation for these data is that ambient concentrations of plasma free linoleic and arachidonic acids act positively via concentration dependent reactions to increase the rate of DNA synthesis in tumours cells that are in S phase. Clearly, these early events in linoleate- and arachidonate-stimulated tumour $^3$H-thymidine incorporation are imperfectly understood and require further experimentation.

It is of interest to compare the specificity of fatty acid requirements in: tumour growth *in vivo* (see Welsch, 1987 for a review); $^3$H-thymidine incorporation in tumours perfused *in situ*; and $^3$H-thymidine incorporation and growth in cancer cells in culture. Similarities and differences discovered using these three different systems are pertinent. Despite early reports that high fat diets increased tumour growth, evidence now points to dietary linoleic acid intake as the critical factor (Hillyard & Abraham, 1979; Ip *et al.*, 1985). Ingested arachidonic acid had no effect (Hillyard & Abraham, 1979). In solid tumours perfused *in situ*, linoleic acid caused a large response in $^3$H-thymidine incorporation; arachidonic, gamma-linolenic and dihomo-gamma linolenic acids were also active, albeit at about one-third the activity of linoleic acid (Sauer & Dauchy, 1988; and see Figure 1 and Table II). Perfusion with saturated or monounsaturated fatty acids had no effect (Sauer & Dauchy, 1988). Rodent and human tumour cells in culture show increased rates of cell proliferation (Holley *et al.*, 1974; Wicha *et al.*, 1979; Rose & Connelly, 1990) and increased rates of $^3$H-thymidine incorporation (Wicha *et al.*, 1979; Rose & Connelly, 1990) when linoleic, arachidonic or oleic acid was included in the medium. Thus, the fatty acid requirement for growth of tumours *in vivo*, for $^3$H-thymidine incorporation in tissue-isolated perfused tumours, or for $^3$H-thymidine incorporation and growth of tumour cells in culture is characterised by a progressive decrease in fatty acid specificity. Studies of mechanism require models that duplicate the intact animal; we believe that the tissue-isolated tumour perfused *in situ* serves this purpose best.

The mechanism of action of the omega-3 fatty acids is also not known. Ingestion of these fatty acids has an inhibitory effect on tumour growth *in vivo* (Karmali *et al.*, 1984; Gabor & Abraham, 1986) and, when omega-3 fatty acids were ingested with linoleic acid, the positive growth effects of linoleic acid were decreased (Gabor & Abraham, 1986). Experiments reported here show that alpha-linolenic and eicosapentaenoic acids inhibited both linoleic acid uptake and the stimulative effect of linoleate on tumour $^3$H-thymidine incorporation (Table III and Figure 2). Assuming that inhibition of $^3$H-thymidine incorporation in a tumour perfused *in situ* and inhibition of growth of a tumour mass in the intact rat are the first and the final stages of the same inhibitory process, one could expect similar mechanisms. In support of this reasoning, it is known that feeding diets containing omega-3 fatty acids either alone or in combination with linoleic acid caused a substantial decrease in the tumour content of linoleic acid (Gabor & Abraham, 1986), suggesting a decrease in uptake. It seems likely, therefore, that ingestion of dietary oils containing omega-3 fatty acids increased the ambient levels of plasma free omega-3 fatty acids in host arterial blood and this increase competively inhibited tumour linoleate uptake. Since linoleic acid uptake appears to be critical for determining the tumour growth rate, the result is an omega-3 fatty acid-mediated decrease in tumour growth. Further experiments designed to test this hypothesis are in progress.

This research was supported by Grant No. CA27809-11 from the National Cancer Institute, NIH and Grant No. 90A42 from the American Institute for Cancer Research. We wish to thank Dr Estelle Goodell and Heidi Johnson for help in preparing the Tables.

### References

ABRAHAM, S. & HILLYARD, L.A. (1983). Effects of dietary 18-carbon fatty acids on growth of transplantable mammary tumors in mice. *J. Natl Cancer Inst.*, **71**, 601–605.

DOUMAS, B.T. & BIGGS, H.G. (1972). Determination of serum albumins. In *Standard Methods in Clinical Chemistry*, Cooper, G.A. (ed) p. 175–188, Vol. 7, Academic Press: New York.

GABOR, H., HILLYARD, L.A. & ABRAHAM, S. (1985). Effect of dietary fat on growth kinetics of transplantable mammary carcinoma in BALB/c mice. *J. Natl Cancer Inst.*, **74**, 1299–1305.

GABOR, H. & ABRAHAM, S. (1986). Effect of dietary menhaden oil on tumour cell loss and the accumulation of mass of a transplantable mammary adenocarcinoma in BALB/c mice. *J. Natl Cancer Inst.*, **76**, 1223–1229.

HILLYARD, L.A. & ABRAHAMS, S. (1979). Effect of dietary polyunsaturated fatty acids on growth of mammary adenocarcinomas in mice and rats. *Cancer Res.*, **39**, 4430–4437.

HILLYARD, L.A., RAO, G.A. & ABRAHAMS, S. (1980). Effect of dietary fat on fatty acid composition of mouse and rat mammary adenocarcinomas. *Proc. Soc. Exp. Biol. Med.*, **163**, 376–383.

HOLLEY, R.W., BALDWIN, J.H. & KIERNAN, J.A. (1974). Control of growth of a tumour cell by linoleic acid. *Proc. Natl Acad. Sci. USA*, **71**, 3976–3978.

HOLMAN, R.T. (1960). The ratio of trienoic:tetraenoic acids in tissue lipids as a measure of essential fatty acid requirement. *J. Nutrit.*, **70**, 405–410.

IP, C., CARTER, C.A. & IP, M.M. (1985). Requirement of essential fatty acid for mammary tumorigenesis in the rat. *Cancer Res.*, **45**, 1997–2001.

JURKOWSKI, J.J. & CAVE, W.T. (1985). Dietary effects of menhaden oil in the growth and membrane lipid composition of rat mammary tumors. *J. Natl Cancer Inst.*, **74**, 1145–1150.

KARMALI, R.A., MARSH, J. & FUCHS, C. (1984). Effect of omega-3 fatty acids on growth of a rat mammary tumour. *J. Natl Cancer Instit.*, **73**, 457–461.

NESTEL, P.J. & STEINBERG, D. (1963). Fate of palmitate and of linoleate perfused through the isolated rat liver at high concentrations. *J. Lipid Res.*, **4**, 461–469.

ROGERS, A.E. & WETSEL, W.C. (1981). Mammary carcinogenesis in rats fed different amounts and types of fat. *Cancer Res.*, **41**, 3735–3737.

ROSE, D.P. & CONNOLLY, J.M. (1990). Effects of fatty acids and inhibitors of eicosanoid synthesis on the growth of a human breast cancer cell line in culture. *Cancer Res.*, **50**, 7139–7144.

SAUER, L.A., STAYMAN, J.W. & DAUCHY, R.T. (1982). Amino acid, glucose, and lactic acid utilization *in vivo* by rat tumours. *Cancer Res.*, **42**, 4090–4097.

SAUER, L.A., NAGEL, W.O., DAUCHY, R.T., MICELI, L.A. & AUSTIN, J. (1986). Stimulation of tumour growth in adult rats *in vivo* during an acute fast. *Cancer Res.*, **46**, 3469–3475.

SAUER, L.A. & DAUCHY, R.T. (1988). Identification of linoleic and arachidonic acids as the factors in hyperlipemic blood that increase $^3$H-thymidine incorporation in hepatoma 7288CTC perfused *in situ*. *Cancer Res.*, **48**, 3106–3111.

SAUER, L.A. & DAUCHY, R.T. (1900). Tumour-host metabolic interrelationships. *Biochem. Soc. Trans.*, **18**, 80–82.

SAUER, L.A. & DAUCHY, R.T. (1992). Uptake of plasma lipids by tissue-isolated hepatomas 7288CTC and 7777 *in vivo*. *Br. J. Cancer*, **66**, 290–296.

SOLER-ARGILAGA, C., INFANTE, R., RENAUD, G. & POLONOVSKI, J. (1974). Factors influencing free fatty acid uptake by the isolated perfused rat liver. *Biochimie*, **56**, 757–761.

SPECTOR, A.A. & HOAK, J.C. (1969). An improved method for the addition of long-chain free fatty acids to protein solutions. *Anal. Biochem.*, **32**, 297–302.

TRAVIS, J. & PANNEL, R. (1973). Selective removal of albumin from plasma by affinity chromatography. *Clin. Chem. Acta.*, **49**, 49–52.

VAN HARKEN, D.R., DIXON, C.W. & HEIMBERG, M. (1969). Hepatic lipid metabolism in experimental diabetes. V. The effect of concentration of oleate on metabolism of triglycerides and on ketogenesis. *J. Biol. Chem.*, **244**, 2278–2285.

WELSCH, C.W. (1987). Enhancement of mammary tumorigenesis by dietary fat: review of potential mechanisms. *Am. J. Clin. Nutr.*, **45**, 192–202.

WICHA, M.S., LIOTTA, L.A. & KIDWELL, W.R. (1979). Effects of free fatty acids on the growth of normal and neoplastic rat mammary epithelial cells. *Cancer Res.*, **39**, 426–435.